# An information theory approach to biocultural complexity

**DOI:** 10.1038/s41598-020-64260-5

**Published:** 2020-04-29

**Authors:** M. Humberto Reyes-Valdés, Stella K. Kantartzi

**Affiliations:** 1 Universidad Autónoma Agraria Antonio Narro, Graduate Program on Plant Genetic Resources for Arid Lands, Saltillo, Coahuila 25315 Mexico; 2 Southern Illinois University, Department of Plant, Soil and Agricultural Systems, Carbondale, IL 62901 USA

**Keywords:** Computational biology and bioinformatics, Ecology, Plant sciences

## Abstract

The study of biocultural diversity requires the use of appropriate concepts and analytical tools. Particularly, there is a need of indices capable to show the degree of stratification in the set of interactions among cultures and groups of plants and animals in a given region. Here, we present a mathematical approach based on the mutual Shannon information theory to study the relationships among cultural and biological groups. Biocultural complexity was described in terms of effective biocultural units, a new concept defined in this work. From the mathematical formulation of biocultural complexity, formulas were derived to measure the specificity of biological groups and the specialization of cultures, based on the association of human societies with plant or animal groups. To exemplify the concepts and tools, two data sets were analyzed; 1) a set that included artificial data in order to demonstrate the use of the formulas and calculate the indices, and 2) a set that included published data on the use of 18 mushroom species by people in five villages of eastern India. Analysis of the first data set revealed a clear case of biocultural complexity, whereas that of the second set showed that the villages and the use of biological resources composed a single biocultural unit. Overall, hypothesis testing of the association among cultures and biological species was consistent with the information that was provided by the new indices.

## Introduction

Biocultural diversity is a relatively new concept, which can be defined as the biological, cultural, and linguistic diversity, including all the interrelationships, within a complex socio-ecological adaptive system^1,2^. Previous studies on biocultural diversity were transdisciplinary and comprised linguistic, cultural, and biological research methods along with the use of several statistical and mathematical approaches. Diversity in cultures and languages is related to variations in plants, animals, and microorganisms; cultures have evolved in biological contexts, whereas languages contain informational patterns about the biological variation of the surrounding environment and especially, the use of biodiversity. Thus, cultural diversity has been considered as linked to biological variation^1^. The cultural elements that interrelate with biodiversity include religions and ethnic groups, as well as manifestations that extend beyond languages, such as local knowledges, world views, governances and livelihoods. While the number of languages, religions and ethnic groups in a geographic are key to cultural diversity, beliefs, cultural values and worldviews are integrated with a sustainable biological resource mangement^3^. It has been reported that language bears the stamp of the physical environment in which the speakers are placed, while it reflects the interest of people on such environmental features^4^. The existence of hot spots of biocultural diversity^5^ depends on historical, climatic, and geophysical factors. For instance, biological and cultural diversity tends to be high in mountain regions^6^. Social factors, such as human migration, may also impact biodiversity directly^7^. Although language diversity correlates with biodiversity, it is so with climatic variables. A global analysis showed that environmental factors are determinant for the distribution of the diversity of human languages across the world, along with variation in biodiversity^8^. However, tools and concepts are needed to investigate the direct relationships between cultures and biological elements.

In view of the loss of cultural and biological diversity around the globe^9^, attempts have been made to either measure biocultural diversity, or correlate both kinds of diversity^5^. The central problem is that such approaches do not consider the connections between elements in both sides in a given location, where several cultural elements interact with nonhuman biological species. Thus, they miss the structure of the ensemble. For instance, a measure that just adds up both types of diversity in a given region, will not change with stratification in the uses of plants species across the groups that share the territory. On the other hand, correlations between cultural and biological diversity across locations, will not change with the structure of the interrelations inside locations. This creates a gap of knowledge that can impact management strategies, because a given region may, erroneously, be considered as a uniform system of interrelations, and not a structured one.

Global data need to be analyzed using information and communication technologies to identify any inferential and causal relationships between biological and cultural systems. Only a few studies have focused on the interdisciplinary and transdisciplinary connections of the biological and cultural diversity and consequently, analytical tools are limited^2^. To provide objective measures, a universal index of biocultural diversity (IBCD) that is based on the number of languages, religions, ethnic groups, and non-human biological species in a specific region, has been proposed^5^, which is described by Eq. (1):

1 $$IBCD=\frac{CD+BD}{2},$$

where $CD$ is the value of cultural diversity and $BD$ is the value of biological diversity. The values $CD$ and $BD$ are defined as a function of the logarithm of the number of cultural and biological units, respectively^5^. However, $IBCD$ assumes additivity of the two types of diversity but ignores the association of social categories with certain biological species. Other indices have been proposed for defining the biological or cultural components of a biocultural system, e.g. the index of linguistic diversity based on the geometric mean^10^.

Attempts have been made to infer the association between cultural and biological diversity. It is known that low biological diversity is associated with low cultural diversity and that the loss of languages is accompanied by the loss of plant and animal species (*i.e*., co-occurrence of biological and linguistic diversity). Pearson and Spearman correlations have been used to infer linear associations between the number of languages and that of vascular plant species^11^. The results showed positive, albeit low correlations. This statistical approach allows the detection of linear correlations between biological and cultural diversity; however, it fails to detect the associations or the structure of the relationships among cultural groups and plant or animal species within a specific region, precluding the possibility to quantify the biocultural complexity.

Conversely to approaches that in an additive fashion evaluate cultural and biological diversity, or those that estimate the correlation between both instances, what we propose here is a means to gain insights about the structure of local biocultural diversity, by the use of an indicator that is sensitive to connections between the elements of the cultural and biological ensembles, to measure what we call biocultural complexity. In general terms, the more structured a biocultural system, the greater its complexity. Although there is no consensus about what complexity is and its definition depends of the case being studied, a well-accepted conceptual approach is to define it through information metrics based on Shannon entropy^12,13^.

Information theory is basically the study of the blocks of a communication channel. It was pioneered by Claude Shannon^14^, and allows among many other applications, to quantify information. Although this branch of mathematics was originally applied to electrical communications, the solidity of its foundations and universality of its concepts has allowed applications to many fields. The concept of entropy is central in information theory. It is a measure of uncertainty, that has been applied to measure complexity in a system^12,13^. In ecology, the Shannon entropy is a well-known measure of species diversity, which has been called Shannon diversity^15^. The Shannon entropy concept is the basis of the definition and measure of information as a reduction of uncertainty. Along this paper, we use the term mutual information, which is current in the mathematical theory, as the measure of reduction of uncertainty in the value of a variable, given knowledge of the value of another variable. Mutual information has several properties, one of the most notorious being its symmetry^16^.

In the last decades, information theory has been widely applied to biological areas. For instance, it has been used as a mathematical approach to molecular biology^17^. It is a tool for sequence analysis in bioinformatics^18^. It has been applied for measuring and optimizing genetic diversity^19,20^, and the study of transcriptomes and its relationship with cancer^21,22^. Since it studies information storage, transmission, and recovery, it could be used to gain insights into the society and nature from an informational point of view^23^.

Based on our current knowledge on the diversity, management, history, and geography of human societies, mutual information among social features and plant or animal species is expected to be significant. Therefore, the application of information theory for assessing the biocultural diversity of a specific region may reveal new relationships among cultures, languages, and biological species.

The herein proposed concept of biocultural complexity originates two additional new indicators: specificity of biological groups and specialization of cultural units, analogous to previously described indices for transcriptome analysis^21^. The indices and formulas were applied for the analysis of an artificial data set as well as a set of published data^24^.

## Model

### Overview

The general approach of this study was based at the ensemble of cultures and biological species as two associated entities that contain information about each other. A human group was defined as a set of cultures, determined by religions, languages, traditions, tribal relationships or geographic areas. Each of these groups was characterized by the interaction with a subset of a defined set of plant or animal species. The intensity of the association between the set of cultures and the subsets of biological species was measured by the mutual information. A zero value for mutual information would be the extreme case, in which the set of cultures was homogeneous for the interaction with the biological species; thus, the ensemble was composed by a single biocultural unit. The opposite extreme would emerge if each cultural group had an interaction with a unique subset of species; thus, the ensemble was composed by as many biocultural units as cultural groups. Following this approach, a set of indices was defined to evaluate the biocultural complexity of a specific region. Two of these indices had the same mathematical representation as previously ones devised by the use of an information theory approach for the study of transcriptomes^21^.

### Mutual information and biocultural complexity

A set of $c$ cultural groups distributed in a specific region was defined and characterized by the interaction with $s$ biological species ($s\ge c$). As interaction could be considered for instance the use of plants as food, medicine or elements of religious ceremonies. If ${f}_{ij}$ is the frequency of the association between the *i*-$th$ species and the *j*-$th$ culture, the mutual information between cultures and species is defined as follows:

2 $$I(S;C)={H}_{C}-{H}_{C|S}={H}_{S}-{H}_{S|C},$$

where ${H}_{C}$ and ${H}_{S}$ are Shannon entropies^14^ of cultures and species, respectively. ${H}_{C|S}$ and ${H}_{S|C}$ are the conditional entropy of cultures for specific species and of species for specific cultures, respectively^16^. From the first expression of the right side of Eq. (2), the mutual information could be expressed as a function of frequencies (Supplementary Material):

3 $$I(S;C)=-\,\mathop{\sum }\limits_{j=1}^{c}\,{f}_{\mathrm{}.j}lo{g}_{2}({f}_{\mathrm{}.j})+\mathop{\sum }\limits_{i=1}^{s}\,\mathop{\sum }\limits_{j=1}^{c}\,{f}_{ij}lo{g}_{2}({f}_{j|i}),$$

where ${f}_{.j}$ is the marginal frequency of the *j*–*th* culture, calculated by ${f}_{.j}={\sum }_{i=1}^{s}\,{f}_{ij}$. The symbol ${f}_{j|i}$ is the conditional frequency of the *j*–*th* culture given the *i*–*th* species. Equation (3) can be interpreted as the average information about the identity of cultures for an associated species or as the average information about species given an associated culture. It measures how much do we know about the identity of the associated members of a group of cultures, by knowing the identity of a given biological species. The adjective mutual comes form the symmetry of this mathematical formula, because it also measures how much do we know about the identity of the associated biological species, by knowing the identity of a given cultural group. This symmetrical behavior is a general mathematical property of information defined through the Shannon entropy^16^. The minimum value is zero, when all cultures interact evenly with biological species, whereas the maximum value is *log*_2_(*c*), when each culture interacts with a private set of species (Supplementary Material). If each cultural group is evenly represented in the region of study, the marginal frequencies of cultures are equal: ${f}_{.1}={f}_{.2}=\cdots ={f}_{.c}=1/c$. Therefore Eq. (3) could be expressed as follows:

4 $$I(S;C)=lo{g}_{2}(c)+\frac{1}{c}\,\mathop{\sum }\limits_{i=1}^{s}\,\mathop{\sum }\limits_{j=1}^{c}\,{f}_{i|j}lo{g}_{2}({f}_{j|i}),$$

leading to an index we have named biocultural complexity:

5 $$BC={2}^{I(S;C)},$$

and interpreted as the number of effective biocultural units. The number of effective biocultural units is defined as the number of cultures, each associated with an exclusive or private set of biological species, that would exhibit the same mutual information as the actual ensemble of cultures and species. This index is ranged from 1 to $c$ (Supplementary Material). The minimum value, $BC=1$, is attained when all cultures share the same species in equal proportions, and thus, the ensemble can be considered as a unique biocultural unit. The maximum, $BC=c$, occurs when each culture interacts with a private set of species, and thus, the ensemble is composed by $c$ biocultural units. Another interpretation of the biocultural complexity, as defined by Eq. (5), is the ratio between species and species within groups diversity using of the exponential Shannon entropy, which has the intuitive properties of diversity metrics^15^. Furthermore, if the cultural groups exist in an equiprobable space, $BC$ is the ratio of the number of cultural groups and the exponential Shannon diversity of cultural groups for specific species (Supplementary Material).

One can see biocultural complexity as a measure of how structured is a biocultural ensemble. Although it is an abstract concept, our measure is scored by the effective number of biocultural units, in an analogous way to a well-known concept in population genetics, called effective size of a population, which can be defined as the size of an idealized population that would have the same homozygosity increase as the actual population^25^. Following this simile, the idealized biocultural ensemble would be the most structured one, in which every cultural group interacts with a private set of biological species. While in almost all situation this would not be the case, the numeric value of biocultural complexity will indicate the size of the ideal biocultural ensemble whose complexity equals to the actual one.

### Specificity of biological species

A measure of how specific is a biological group in its relationships with a set of cultural groups was defined as follows:

6 $$\begin{array}{rcl}{S}_{i} & = & H(C)-H(C|{S}_{i})\\  & = & -\,\mathop{\sum }\limits_{j=1}^{c}\,{f}_{\mathrm{}.j}lo{g}_{2}({f}_{\mathrm{}.j})+\mathop{\sum }\limits_{j=1}^{c}\,{f}_{j|i}lo{g}_{2}({f}_{j|i}),\end{array}$$

where ${f}_{.j}$ is the marginal frequency of the ${j}_{th}$ cultural group, and ${f}_{j|i}$ is the conditional frequency of the *j*–*th* cultural group for the *i*–*th* species, which can be calculated by the Bayes theorem as follows:

$${f}_{j|i}=\frac{{f}_{i|j}{f}_{.j}}{{\sum }_{k=1}^{c}\,{f}_{i|k}{f}_{.k}}=\frac{{f}_{ij}}{{f}_{i.}}$$

The index ${S}_{i}$ is bounded by 0 and *log*_2_(*c*). A zero value for taxon specificity indicates that the given biological group has a uniform interaction across cultural groups, whereas the maximum value *log*_2_(*c*) indicates that the given species is private of a given cultural group. From the informational point of view, ${S}_{i}$ measures the amount of information that a species carries about the identity of the cultural groups that interacts with.

If each cultural group is evenly represented in the region of study then ${S}_{i}$ can be expressed as follows (Supplementary Material):

7 $${S}_{i}=\frac{1}{c{f}_{i\mathrm{}.}}\,\mathop{\sum }\limits_{j=1}^{c}\,{f}_{i|j}lo{g}_{2}\left(\frac{{f}_{i|j}}{{f}_{i\mathrm{}.}}\right)$$

where ${f}_{i\mathrm{}.}$ is the average frequency of the *i*–*th* species within cultural groups. Equation (7) is equivalent to the allele specificity for transcriptome analysis^21^.

### Specialization of cultural groups

The specialization of the *j*–*th* cultural group was defined as the weighted average of specificities of the related taxonomic groups:

8 $${\delta }_{j}=\mathop{\sum }\limits_{i=1}^{s}\,{f}_{i|j}{S}_{i}$$

The specialization index ${\delta }_{j}$ is bounded by 0 and *log*_2_(*c*). The value of zero is attained when all associated species to the *j*–*th* cultural group have a zero specificity. The maximum value of *log*_2_(*c*) is attained when all associated species are private of the given cultural group. Equation (7) is equivalent to the tissue specialization derived from transcriptome analysis^21^.

The following equality can be proved (Supplementary Material):

$$I(S;C)=\mathop{\sum }\limits_{i=1}^{s}\,{f}_{i.}{S}_{i}=\mathop{\sum }\limits_{j=1}^{c}\,{f}_{.j}{\delta }_{j},$$

When ${f}_{.1}={f}_{.2}=\cdots ={f}_{.c}$, i.e. when the cultural groups are equiprobable or non-weighted, the equality becomes:

9 $$I(S;C)=\mathop{\sum }\limits_{i=1}^{s}\,{f}_{i.}{S}_{i}=\frac{1}{c}\,\mathop{\sum }\limits_{j=1}^{c}\,{\delta }_{j.}$$

The calculation proceeds as follows: (i) ${S}_{i}$ is calculated for each species through Eq. (6) for data with weighted cultural groups or Eq. (7) for unweighted cultural groups, (ii) ${\delta }_{j}$ is calculated for each cultural group using Eq. (8), (iii) $I(S;C)$ is calculated using the first or second term of Eq. (9), and (iv) $BC$ is calculated using Eq. (5). An *R* application has been developed to perform these analyses, publicly available at the GitHub site https://github.com/mathgenome/biocultural.

Although either ${S}_{i}$ or ${\delta }_{j}$ are calculated as a part of the mechanics to estimate $BC$, we remark that, conceptually, the biocultural complexity is not *a priori* a function of specificities or specializations, but it is rooted on the mutual information between cultural groups and biological species. The mechanics of calculation based on specificities or specializations results from the mathematical properties of $BC$.

Calculations based on the Shannon entropy formula through estimated frequencies, can lead to significant bias with small sample sizes^26^. Since the definition of mutual information in Eq. (2) and thus its associated definitions of biocultural complexity, specificity and specialization, are based on the Shannon entropy, estimations of these parameters are prone to bias, which can be important if small sample sizes are used. To circumvent this problem, we used a bootstrap approach to bias correction. The bootstrap is a resampling method^27^, primarily to estimate standard errors and confidence intervals. As an extended application, a detailed discussion about its application to bias correction can be found^28^. Let $\hat{\theta }$ be the original estimate of a given parameter from the sample, and $\bar{\hat{\theta }}$ the mean of *b* estimates obtained from *b* random samples extracted with replacement from the original sample. The bias was estimated as $\bar{\hat{\theta }}-\hat{\theta }$; then, the estimated bias was substracted from the original sample estimate^28^. For biocultural complexity, a confidence interval was given by^28^: $Prob(2\hat{\theta }-{\hat{\theta }}_{H} < \theta  < 2\hat{\theta }-{\hat{\theta }}_{L})=1-\alpha$, where ${\hat{\theta }}_{L}$ is the 100*α*/2 percentile and ${\hat{\theta }}_{H}$ is the 100(1 − *α*/2) percentile in the bootstrap distribution. The standard errors of the statistics were estimated through the standard deviations of their bootstrap estimates. The number of samples for bootstrap estimations was 1000, which can be considered reliable for both bias and confidence intervals^28^.

## Applications

### Set of artificial data

In the artificial data set, the frequencies of five cultural groups were undefined, and their representation was assumed to be uniform (an equiprobable set). Thus, those frequencies were coded as 1, a suitable format for the developed *R* application. The counts of five species within each cultural group, the conditional frequencies of species, and the normalized frequencies of cultural groups are in Table 1. The relative specificities and relative specializations were calculated by dividing the corresponding value by the maximum theoretical value (*log*_2_(*c*)).

**Table 1:** Artificial data for five species associated to four cultural groups.

Culture	Representation	sp1	sp2	sp3	sp4	sp5
c1	1	0	25	39	27	9
c2	1	110	10	0	0	0
c3	1	0	26	24	28	10
c4	1	0	11	119	0	0
c1	0.25	0.00	0.25	0.39	0.27	0.09
c2	0.25	0.92	0.08	0.00	0.00	0.00
c3	0.25	0.00	0.29	0.27	0.32	0.11
c4	0.25	0.00	0.09	0.92	0.00	0.00

The upper part of the table shows field counts for species, whereas the lower part shows frequencies.

The species sp1 was present in only one cultural group, providing a high specialization weight, and thus, it was expected to be on the upper extreme of specificity. However, the species sp2 was present in all cultures, and thus, it was expected to be close to the lower limit of specificity. For cultural groups, c1 interacted with an exclusive species in a high conditional frequency, and thus, a high weight on its specialization was expected. The specificity statistics of the taxa are presented in Table 2; sp1 was highly specific, presenting the maximum possible score and leading to a relative specificity of 1, whereas sp2 was well-represented among the four cultural groups, showing the lowest specificity score. For cultural groups, c2 was the most specialized (Table 3) since it interacted only with two species, of which sp1, the most specific species, was also the most frequent. For groups c1, c3, and c4, the specialization values were similar. An apparent contradiction emerged for c4, which only interacted with two species; however, these were the least specific ones, and thus, the weighted average for the calculation of cultural specialization resulted in a low value. Furthermore, c4 showed one of the lowest Shannon diversities. Therefore, c4 could be described as a low specialized group with a low diversity of interacting species. In cases that bias was detected for either specificity or specialization, it was upward with a low value. Additionally, slight downward bias was detected for Shannon diversity in cultural groups in only two cases.

**Table 2:** Specificity results for the data of Table 1.

Species	Specificity	CorSpec	SE. Spec	RSpec	CorRSpec	SE.RSpec
sp1	2.00	2.00	0.00	1.00	1.00	0.00
sp2	0.22	0.20	0.08	0.11	0.10	0.04
sp3	0.61	0.60	0.04	0.30	0.30	0.02
sp4	1.00	1.00	0.02	0.50	0.50	0.01
sp5	1.01	0.97	0.07	0.50	0.49	0.03

CorSpec = Bias-corrected specificity, SE. Spec = Standard error of specificity, RSpec = Relative specificity, CorRSpec = Bias-corrected relative specificity, SE.RSpec = Standard error of relative specificity.

**Table 3:** Cultural specialization and taxonomic diversity results for the data of Table 1.

Culture	Specia	CorSpecia	SE.Specia	RSpecia	CorRSpecia	SE.RSpecia	SDiv	CorSDiv	SE.SDiv
c1	0.65	0.64	0.04	0.33	0.32	0.02	1.85	1.87	0.06
c2	1.85	1.85	0.05	0.93	0.93	0.02	0.41	0.41	0.09
c3	0.66	0.65	0.05	0.33	0.32	0.02	1.91	1.94	0.05
c4	0.58	0.57	0.05	0.29	0.28	0.02	0.42	0.42	0.09

Specia = Specialization, CorSpecia = Bias-corrected specialization, SE. Specia = Standard error of specialization, RSpecia = Relative specialization; CorRSpecia = Bias-corrected relative specialization, SE.RSpecia = Standard error of relative specialization, SDiv = Shannon diversity, CorSDiv = Bias-corrected Shannon diversity, SE.SDiv = Standard error of Shannon diversity.

The bias-corrected biocultural complexity was 1.90 with an uncorrected estimate of 1.91, showing that the biocultural ensemble was equivalent to 1.90 totally different biocultural units. The respective 95% confidence interval ranged from 1.81 to 1.99. A chi square test with Monte Carlo simulation for the counting data resulted in a highly significant association between species and cultures with *P* = 0.0005, revealing that the ensemble was equivalent to more than one biocultural unit.

### Set of published data

**Figure 1:**
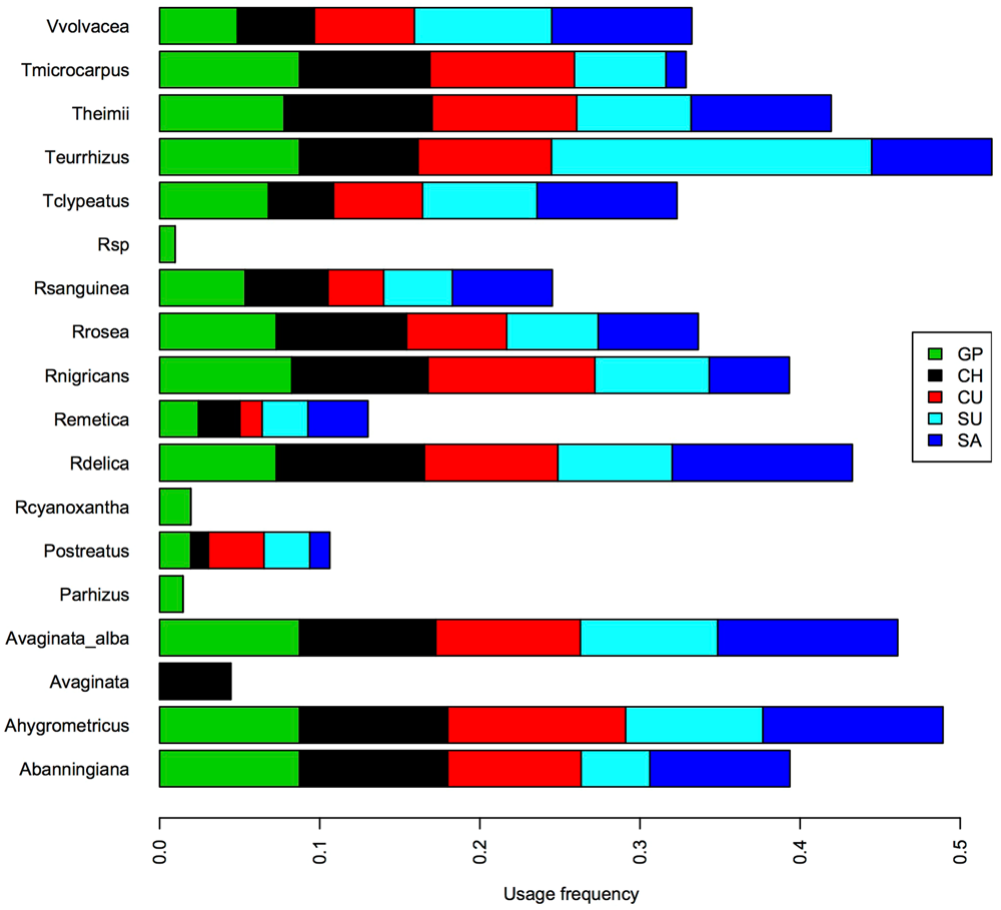
Usage frequency of 18 mushroom groups by the inhabitants of five villages in the eastern lateritic part of India (Manna *et al*.^24^). The meanings of abbreviations for mushroom species are described in Table 4, whereas those for villages are in Table 5.

The set of published data^24^ included counts for 18 mushroom species related to their usage in five villages of eastern India. The villages were equally represented geographically-defined human groups since the cited study did not assign weights for those units. The average mushroom count per village was 154. Figure 1 shows the relative mushroom usage for each village and species (abbreviations provided in Tables 4 and 5). Of the included species, *Russula sp*., *Russula cyanoxantha* and *Pisolithus arhizus* were represented only in one village, Gonpur, and had a very low usage frequency, whereas *Amanita vaginata* is represented only in Choupahari. All other mushroom species were represented in the four villages, and in most cases they were evenly represented, revealing a low biocultural complexity of the ensemble of human communities and mushroom species.

**Table 4:** Specificity results for the Indian mushroom data^24^.

Species	Abbreviation	Specificity	CorSpec	SE.Spec	RSpec	CorRSpec	SE.RSpec
Amanita banningiana	Abanningiana	0.04	0.00	0.07	0.02	0.00	0.03
Amanita vaginata	Avaginata	2.32	2.32	0.00	1.00	1.00	0.00
Amanita vaginata var. alba	Avaginata_alba	0.01	0.00	0.04	0.00	0.00	0.02
Astraeus hygrometricus	Ahygrometricus	0.01	0.00	0.04	0.00	0.00	0.02
Pisolithus arhizus	Parhizus	2.32	2.32	0.00	1.00	1.00	0.00
Pleurotus ostreaeus	Postreatus	0.13	0.05	0.12	0.06	0.02	0.05
Russula cyanoxantha	Rcyanoxantha	2.32	2.32	0.00	1.00	1.00	0.00
Russula delica	Rdelica	0.02	0.00	0.05	0.01	0.00	0.02
Russula emetica	Remetica	0.06	0.00	0.11	0.03	0.00	0.05
Russula nigricans	Rnigricans	0.04	0.00	0.06	0.02	0.00	0.03
Russula rosea	Rrosea	0.01	0.00	0.06	0.01	0.00	0.02
Russula sanguinea	Rsanguinea	0.03	0.00	0.08	0.01	0.00	0.03
Russula sp.	Rsp	2.32	2.32	0.00	1.00	1.00	0.00
Termitomtces heimii	Theimii	0.01	0.00	0.05	0.00	0.00	0.02
Termitomyces clypeatus	Tclypeatus	0.04	0.00	0.07	0.02	0.00	0.03
Termitomyces eurrhizus	Teurrhizus	0.13	0.09	0.09	0.06	0.04	0.04
Termitomyces microcarpus	Tmicrocarpus	0.18	0.16	0.07	0.08	0.07	0.03
Volvaria volvacea	Vvolvacea	0.05	0.00	0.07	0.02	0.00	0.03

CorSpec = Bias-corrected specificity, SE.Spec = Standard error of specificity, RSpec = Relative specificity, CorRSpec = Bias-corrected relative specificity, SE.RSpec = Standard error of relative specificity.

**Table 5:** Cultural specialization and taxonomic diversity results for the Indian mushroom data^24^.

Village	Abbr	Specia	CorSpecia	SE.S	R. Specialization	CorRSpecia	SE.RS	S. Diversity	CorSDiver	SE.SD
Gonpur	GP	0.15	0.10	0.03	0.07	0.04	0.01	3.88	4.01	0.03
Choupahari	CH	0.15	0.19	0.02	0.06	0.08	0.01	3.77	3.93	0.04
Curicha	CU	0.05	0.00	0.03	0.02	0.00	0.02	3.68	3.53	0.04
Sultanpur	SU	0.06	0.02	0.02	0.03	0.01	0.01	3.62	3.69	0.06
Sarmara	SA	0.04	0.00	0.03	0.02	0.00	0.01	3.63	3.74	0.09

Abbr = Village abbreviature, Specia = Specialization, CorSpecia = Bias-corrected specialization, SE.Specia = Standard error of specialization, RSpecia = Relative specialization; CorRSpecia = Bias-corrected relative specialization, SE.RSpecia = Standard error of relative specialization, SDiv = Shannon diversity, CorSDiv = Bias-corrected Shannon diversity, SE.SDiv = Standard error of Shannon diversity.

The results for the 18 species are summarized in Table 4. The maximum specificity was attained by *Amanita vaginata*, *Pisolithus arhizus*, *Russula cyanoxantha*, and *Russula sp*., with a value of 2.32, which equals the theoretical maximum with a corresponding relative value of 1. This value was consistent with the fact that each of the four species was private for a single village. All other species showed specificities close to 0 with a slight upward bias. The biocultural specializations and taxonomic diversities for all five villages are presented in Table 5. The specializations were very low for the five inhabited sites with a maximum value of 0.19, attained by Choupahari. Four of the raw estimates showed an upward bias with the exception of Choupahari specialization, which exhibited a downward bias of 0.04. All cultural groups shared similar taxonomic diversity indices; four raw estimates showed a small downward bias, whereas one showed a slight upward bias. These results revealed an ensemble of villages with low biocultural complexity. In fact, the bias-corrected biocultural complexity was estimated at 1.02 with a 95% confidence interval ranging from 1 to 1.05, indicating that the set of villages could be considered as a single biocultural unit. Such a conclusion was also supported by the chi square test with Monte Carlo simulation for the relationship between villages and mushroom usage, which resulted in a non-significant *P* = 0.1159, indicating homogeneity among sites. The non-corrected point estimate of biocultural complexity was 1.06 with an upward bias of 0.04.

## Discussion

The information theory-based parameters proposed in the present study provided a theoretical approach for the analysis and quantification of biocultural complexity (*BC*). It allowed calculation of taxa specificity, as an index of the uniqueness level of taxonomic units with reference to cultural groups. Furthermore, it set the basis for calculation of cultural specialization, which depends on the frequency and specificity of the taxonomic units that compose the biological interaction structure of a cultural group. The frequency of specific plants or animals that interact with a given group increases with the specialization of the group. The specificity and specialization indices have a direct mathematical relationship with the biocultural complexity, which can be interpreted as the effective number of biocultural units equivalent to the observed data.

One of the implications of a high *BC* in a local ensemble of cultures and biological species is the presence of structure, which indicates differential frequencies of species usage among cultures. One issue is the possible occurrence of different ways of use among the cultural groups for a given species, $e\mathrm{}.g\mathrm{}.$ some groups may use it only as food whereas others use the same species also as medicine. If the data for counts of species usage among groups was collected regardless of the type of use, then *BC* will not be sensitive to any structure of the biocultural ensemble resulting from differential usage type. However, *BC* calculation can be assayed by counts in accordance to ways of species usage. Following our example, if all groups use a species for food and only one for medicine, then *BC* calculation by incorporation of the exclusive use of the species as medicine, will be sensitive to the structure derived from this differential exploitation of a biological resource. Thus, *BC* can aid to understand the role of cultural manifestations such as knowledges and livelihoods in the structure of a local biocultural ensemble.

The artificial data set analyzed in the present study showed that the specificity of taxonomic groups was related to the level of uniqueness in their usage by cultural groups, and also that highly specialized groups were characterized by the frequent use of highly specific taxonomic units. The application of the proposed theory to a data set of mushroom usage in five Indian villages showed that the biocultural ensemble was composed by low specialized groups, and could be considered as a single biocultural unit.

Both the artificial and previously published data sets revealed the consistency of the information-based methods regarding the complexity of ensembles of human groups and the use of biological species. Therefore, the suggested approach allows the quantification of the level of stratification of a biocultural landscape as well as the uniqueness of biological species and cultural groups.

## Supplementary information

Supplementary Information.

## Data Availability

The *R* code and additional tools to use the methods and repicate the analyses are available at the GitHub site https://github.com/mathgenome/biocultural.
