# Supplementary material for: An information theory approach to biocultural complexity
Source: Sci Rep. 2020 Apr 29;10:7203. doi: 10.1038/s41598-020-64260-5 (PMC7190823; doi:10.1038/s41598-020-64260-5)
Supplement: Supplementary file 1 — Supplementary Information. [file 41598_2020_64260_MOESM1_ESM.pdf]

An information theory approach to biocultural complexity

Supplementary file. Mathematical addendum

M. Humberto Reyes-Valdés[1] and Stella K. Kantartzi[2]

[1]Universidad Autónoma Agraria Antonio Narro, Graduate Program on Plant Genetic Resources for Arid Lands, Saltillo, Coahuila 25315, Mexico
[2]Southern Illinois University, Department of Plant, Soil and Agricultural Systems, Carbondale IL, 62901 USA

Mutual information

In Eq (2) we have the mutual information between cultures and species:

$$I(S;C) = H_C - H_{C|S} = H_S - H_{S|C}$$

The entropy of the cultural groups H_C is:

$$H_C = -\sum_{j=1}^{c} f_{.j} log_2(f_{.j}),$$

since the frequency of the $i-th$ group is $f_{.j}$. The conditional entropy of cultural groups given species $H_{C|S}$ can be written as:

$$H_{C|S} = -\sum_{i=1}^{s}\sum_{j=1}^{c} f_{ij} log_2(f_{j|i}),$$

where f_{ij} is the frequency of the combination of the $i-th$ species with the $j-th$ cultural group, and $f_{j|i}$ is the conditional frequency of the $j-th$ cultural group given the $i-th$ species. Thus, Eq. (2) can be written as:

$$I(S;C) = -\sum_{j=1}^{c} f_{.j} log_2(f_{.j}) + \sum_{i=1}^{s}\sum_{j=1}^{c} f_{ij} log_2(f_{j|i})$$

The minimum value of $I(S;C)$ can be easily derived from the second term of the right side of Eq. (2). It occurs when $H_S = H_{S|C}$, i.e. when the global species frequencies are the same as the frequencies within cultural groups, with $I(S;C) = 0$.

The maximum value of $I(S;C)$ can be derived from the first term of the right side of Eq. (2). It occurs when H_C reaches its maximum and $H_{C|S}$ reaches its minimum. The maximum value of H_C is $log_2(c)$, when the frequencies of cultural groups are all equal to $1/c$. The minimum value of $H_{C|S}$ is reached when $f_{j|i} = 1$ for all i, j, i.e. when each species is associated to a unique cultural group, thus $H_{C|S} = 0$ and the maximum value of $I(S;C)$ is $log_2(c)$. To summarize this in a more formal way, assume that $f_{.j} = 1/c$ for all j. Thus:

$$\begin{aligned} I(S;C) &= -\sum_{j=1}^{c} f_{.j} log_2(f_{.j}) + \sum_{i=1}^{s}\sum_{j=1}^{c} f_{ij} log_2(f_{j|i}) \\ &= -c\left(\frac{1}{c} log_2 \frac{1}{c}\right) + \sum_{i=1}^{s}\sum_{j=1}^{c} f_{ij} log_2(f_{j|i}) \\ &= log_2(c) + \sum_{i=1}^{s}\sum_{j=1}^{c} f_{ij} log_2(f_{j|i}) \end{aligned}$$

Now, assume that $f_{j|i} = 1$ for all i, j. To have this equality for all i, j, it is needed that $s \geq c$, as a model requirement stated in the paper. If we add this scenario, we obtain:

$$
\begin{aligned}
I(S;C) &= log_2(c) + \sum_{i=1}^{s}\sum_{j=1}^{c} f_{ij} log_2(f_{j|i}) \\
&= log_2(c) + \sum_{i=1}^{s}\sum_{j=1}^{c} f_{ij} log_2(1) \\
&= log_2(c)
\end{aligned}
$$

The lower and upper bounds of BC

In Eq. (5) biocultural complexity is defined as:

$$BC = 2^{I(S;C)}$$

Since the limits of $I(S;C)$ are $(0, log_2(c))$, the limits of BC are:

$$\left(2^0, 2^{log_2(c)}\right) = (1, c),$$

which are the lower and upper bounds of BC.

Interpretation of BC in terms of species diversity

From Eq (2) BC can be written as follows

$$
\begin{aligned}
BC &= 2^{I(S;C)} \\
&= 2^{(H_S - H_{S|C})} \\
&= \frac{2^{H_S}}{2^{H_{S|C}}},
\end{aligned}
$$

i.e. BC is the ratio between species and species within cultural groups diversity, based on the exponential Shannon entropy (Jost and Baños, 2006).

BC under an equiprobable set of cultural groups

If the marginal frequencies of cultural groups are $f_{.1} = f_{.2} = ... = f_{.c} = 1/c$, then BC can be simplified as follows:

$$
\begin{aligned}
BC &= 2^{I(S;C)} \\
&= 2^{\left[log_2(c) + \sum_{i=1}^{s}\sum_{j=1}^{c} f_{ij} log_2(f_{j|i})\right]} \\
&= \frac{c}{2^{-\sum_{i=1}^{s}\sum_{j=1}^{c} f_{ij} log_2(f_{j|i})}} \\
&= \frac{c}{2^{H_{C|S}}},
\end{aligned}
$$

i.e., if cultural groups are equiprobable, then BC is the ratio between number of cultural groups and culture diversity within species, with diversity defined by the exponential Shannon entropy.

S_i for evenly represented (equiprobable) cultural groups

We start from Eq (6), assuming that $f_{.j} = 1/c$ for all i.

$$\begin{aligned} S_i &= -\sum_{j=1}^{c} f_{.j} log_2(f_{.j}) + \sum_{j=1}^{c} f_{j|i} log_2(f_{j|i}) \\ &= log_2(c) + \sum_{j=1}^{c} f_{j|i} log_2(f_{j|i}) \end{aligned}$$

The conditional probability $f_{j|i}$ can be written as $f_{ij}/f_{i.}$, thus:

$$S_i = log_2(c) + \sum_{j=1}^{c} \frac{f_{ij}}{f_{i.}} log_2 \left(\frac{f_{ij}}{f_{i.}} \right)$$

Since $f_{.j} = 1/c$, $f_{i,j}$ can be written as $f_{i|j}/c$. By substitution we obtain:

$$\begin{aligned} S_i &= log_2(c) + \sum_{j=1}^{c} \frac{f_{i|j}}{cf_{i.}} log_2 \left(\frac{f_{i|j}}{cf_{i.}} \right) \\ &= log_2(c) + \frac{1}{cf_{i.}} \sum_{j=1}^{c} f_{i|j} log_2 \left(\frac{f_{i|j}}{cf_{i.}} \right) \\ &= log_2(c) + \frac{1}{cf_{i.}} \sum_{j=1}^{c} f_{i|j} \left[log_2 \left(\frac{f_{i|j}}{f_{i.}} \right) - log_2(c) \right] \\ &= log_2(c) + \frac{1}{cf_{i.}} \sum_{j=1}^{c} f_{i|j} log_2 \left(\frac{f_{i|j}}{f_{i.}} \right) - \frac{1}{cf_{i.}} \sum_{j=1}^{c} f_{i|j} log_2(c) \end{aligned}$$

Now, $f_{i|j} = cf_{ij}$. Thus:

$$\begin{aligned} S_i &= log_2(c) + \frac{1}{cf_{i.}} \sum_{j=1}^{c} f_{i|j} log_2 \left(\frac{f_{i|j}}{f_{i.}} \right) - \frac{1}{cf_{i.}} \sum_{j=1}^{c} cf_{ij} log_2(c) \\ &= log_2(c) + \frac{1}{cf_{i.}} \sum_{j=1}^{c} f_{i|j} log_2 \left(\frac{f_{i|j}}{f_{i.}} \right) - \frac{c\,[log_2(c)]}{cf_{i.}} \sum_{j=1}^{c} f_{ij} \\ &= log_2(c) + \frac{1}{cf_{i.}} \sum_{j=1}^{c} f_{i|j} log_2 \left(\frac{f_{i|j}}{f_{i.}} \right) - \frac{c\,[log_2(c)]}{cf_{i.}} f_{i.} \\ &= log_2(c) + \frac{1}{cf_{i.}} \sum_{j=1}^{c} f_{i|j} log_2 \left(\frac{f_{i|j}}{f_{i.}} \right) - log_2(c) \\ &= \frac{1}{cf_{i.}} \sum_{j=1}^{c} f_{i|j} log_2 \left(\frac{f_{i|j}}{f_{i.}} \right), \end{aligned}$$

as stated in the paper.

Equality between mutual information and average specificities and specializations

The mutual information between cultures and species can be written as:

$$I(S;C) = H_C - H_{C|S} = \sum_{i=1}^{s} f_{i.} \left[H_C - H_{C|S_i} \right]$$

Thus, we can state from the Eq. (6) in the paper that

$$I(S;C) = \sum_{i=1}^{s} f_{i.} S_i$$

The last result can be worked out as follows:

$$\begin{aligned} I(S;C) &= \sum_{i=1}^{s} f_{i.} S_i \\ &= \sum_{i=1}^{s} \sum_{j=1}^{c} f_{ij} S_i \\ &= \sum_{i=1}^{s} \sum_{j=1}^{c} f_{i|j} f_{.j} S_i \\ &= \sum_{i=1}^{s} f_{.j} \sum_{j=1}^{c} f_{i|j} S_i \\ &= \sum_{i=1}^{s} f_{.j} \delta_j \end{aligned}$$

Thus:

$$I(S;C) = \sum_{i=1}^{s} f_{i.} S_i = \sum_{j=1}^{c} f_{.j} \delta_j$$
